# Multi-method assessment of patients with febrile illness reveals over-diagnosis of malaria in rural Uganda

**DOI:** 10.1186/s12936-016-1502-4

**Published:** 2016-09-07

**Authors:** Ria R. Ghai, Mary I. Thurber, Azza El Bakry, Colin A. Chapman, Tony L. Goldberg

**Affiliations:** 1 Department of Environmental Sciences, Emory University, Atlanta, GA 30322 USA; 2 Department of Biology, McGill University, Montreal, QC H3A 1B1 Canada; 3 School of Veterinary Medicine, University of California Davis, 1 Shields Avenue, Davis, CA 95616 USA; 4 JD Maclean Centre for Tropical Diseases, McGill University, Montreal General Hospital, Montreal, QC H3G 1A4 Canada; 5 Department of Anthropology and McGill School of Environment, McGill University, Montreal, QC H3A 2T7 Canada; 6 Makerere University Biological Field Station, PO Box 967, Kampala, Uganda; 7 Wildlife Conservation Society, 2300 Southern Boulevard, Bronx, NY 10460 USA; 8 Department of Pathobiological Sciences and Global Health Institute, University of Wisconsin-Madison, Madison, WI 53706 USA

**Keywords:** Malaria, *Plasmodium*, Uganda, Rapid diagnostic tests, Misdiagnosis

## Abstract

**Background:**

Health clinics in rural Africa are typically resource-limited. As a result, many patients presenting with fever are treated with anti-malarial drugs based only on clinical presentation. This is a considerable issue in Uganda, where malaria is routinely over-diagnosed and over-treated, constituting a wastage of resources and an elevated risk of mortality in wrongly diagnosed patients. However, rapid diagnostic tests (RDTs) for malaria are increasingly being used in health facilities. Being fast, easy and inexpensive, RDTs offer the opportunity for feasible diagnostic capacity in resource-limited areas. This study evaluated the rate of malaria misdiagnosis and the accuracy of RDTs in rural Uganda, where presumptive diagnosis still predominates. Specifically, the diagnostic accuracy of “gold standard” methods, microscopy and PCR, were compared to the most feasible method, RDTs.

**Methods:**

Patients presenting with fever at one of two health clinics in the Kabarole District of Uganda were enrolled in this study. Blood was collected by finger prick and used to administer RDTs, make blood smears for microscopy, and blot Whatman FTA cards for DNA extraction, polymerase chain reaction (PCR) amplification, and sequencing. The accuracy of RDTs and microscopy were assessed relative to PCR, considered the new standard of malaria diagnosis.

**Results:**

A total of 78 patients were enrolled, and 31 were diagnosed with *Plasmodium* infection by at least one method. Comparing diagnostic pairs determined that RDTs and microscopy performed similarly, being 92.6 and 92.0 % sensitive and 95.5 and 94.4 % specific, respectively. Combining both methods resulted in a sensitivity of 96.0 % and specificity of 100 %. However, both RDTs and microscopy missed one case of non-falciparum malaria (*Plasmodium malariae*) that was identified and characterized by PCR and sequencing. In total, based on PCR, 62.0 % of patients would have been misdiagnosed with malaria if symptomatic diagnosis was used.

**Conclusions:**

Results suggest that diagnosis of malaria based on symptoms alone appears to be highly inaccurate in this setting. Furthermore, RDTs were very effective at diagnosing malaria, performing as well or better than microscopy. However, only PCR and DNA sequencing detected non-*P. falciparum* species, which highlights an important limitation of this test and a treatment concern for non-falciparum malaria patients. Nevertheless, RDTs appear the only feasible method in rural or resource-limited areas, and therefore offer the best way forward in malaria management in endemic countries.

## Background

In Africa, 70 % of fevers are initially managed at home, with traditional remedies and bed rest used to alleviate symptoms [1, 2]. Only when symptoms continue to worsen is medical attention sought, and even at this stage, patients may not receive proper diagnosis—especially at remote or rural health facilities that are often resource-limited [3]. The symptoms of malaria overlap with a number of other illnesses, making accurate diagnosis difficult, even among experienced practitioners [4–7]. In addition, anti-malarial medications can be prescribed by primary care workers who vary in experience and training. As a result, most patients with a recent history of fever are diagnosed with and treated for malaria, despite the fact that a number of other illnesses, including pneumonia, typhoid fever, respiratory tract infections, transient viral illnesses and meningitis may be causing clinical disease [8–10]. Therefore, the sensitivity of malaria diagnosis at clinics may be as high as 100 % since few patients with clinical malaria are missed, but the specificity is often extremely low [5, 6, 11]. Indeed, a study conducted in Tanzanian hospitals showed that less than half of all individuals symptomatically diagnosed for malaria were actually positive for the parasite by microscopic examination of blood smears [12]. Individuals treated for malaria but not actually harbouring the infection were often not prescribed antibiotics, and were more likely to die than individuals with malaria [12]. These results suggest that symptomatic or presumptive diagnosis of malaria is costly not only in terms of wasted anti-malarial drugs, but also in the morbidity and mortality associated with misdiagnosis.

In many malaria endemic regions, safe, inexpensive anti-malarials such as chloroquine have become ineffective due to the emergence of drug-resistant parasites. These drugs are being replaced by more toxic and expensive alternatives, including sulfadoxine-pyrimethamine [13]. Prescribing anti-malarials only following diagnostic testing is perhaps the most appropriate way to dispense drugs, but can be challenging in locations with already overburdened health infrastructure. For instance, the traditional standard of malaria diagnosis is microscopic examination of peripheral blood smears. However, this requires high quality reagents, clean equipment, functioning microscopes, workspace, and skilled personnel [10]. To circumvent poor diagnostic access, antigen-based malaria rapid diagnostic tests (RDTs) have been implemented with success in field conditions, especially in regions without prior access to microscopic diagnostics [3, 14, 15]. Being relatively affordable, easy to use, and quick, RDTs offer a promising method of minimizing over-diagnosis. As a result, there has been a tremendous increase in RDT usage at public health facilities throughout sub-Saharan Africa [2]. However, still only 65 % of suspected malaria cases were diagnostically confirmed by RDT in 2014, and the majority of presumptive diagnoses still occur in sub-Saharan Africa [16].

Uganda is currently among the few countries where cases of malaria have recently increased [16]. Indeed, malaria transmission is endemic across 95 % of the country [17]. This is despite diagnostic testing being free of charge in the public sector, and the recent implementation of the Uganda Malaria Reduction Strategic Plan (2014–2020), which recommends parasite-based diagnosis at all scales and for all patients [16, 18]. One considerable limitation of this plan has been consistent RDT stockouts, which have limited diagnostic capacity, especially in rural areas [19]. Therefore, the objective of this study was to assess the rate of malaria misdiagnosis in a rural area of Uganda when RDT usage was just beginning to circumvent presumptive diagnosis. To establish an accurate estimation of misdiagnosis and assess the efficacy of RDTs, malaria infection was confirmed using two standard diagnostic methods, microscopy and PCR, and the results of RDTs were compared to these methods.

## Methods

### Blood sample collection and processing

Two rural health clinics located within the Kabarole District of Uganda were enlisted in this study. Patients visiting these clinics in June and July 2011 with a fever (an axillary temperature of 37.5 °C or higher), who were not pregnant, and who had not treated their symptoms with standard anti-malarials, were asked to participate. Following informed consent, blood samples were collected via finger prick by the clinic’s health practitioner. One drop of blood was used in an RDT that detected histidine-rich protein II (HRP-II) antigen of *Plasmodium falciparum*, which is the recommended RDT for malaria diagnosis in Uganda. Health practitioners read, interpreted, and recorded the result of the test after 15 min. Results were recorded as positive or negative, with faint lines being interpreted as positive. An additional two drops of blood were used to make thick and thin blood smears for microscopic diagnosis. Finally, one to five additional blood drops were collected on Whatman FTA Classic Cards for subsequent molecular analyses. All patients were offered complimentary treatment based on RDT results and the health practitioner’s diagnosis. Blood samples were shipped to North America for microscopic examination and molecular diagnostics.

### Microscopy

Thin and thick smears were stained with Giemsa and viewed under 100× oil immersion objective magnification. Each slide was read by two independent microscopists; in the event of a discrepancy, the slides were read by a third microscopist. Thick smears were used to confirm the presence of blood parasites, while thin smears were used to confirm speciation and determine infection intensity (parasitaemia). For each thin smear, a field containing a representative monolayer of red blood cells (RBCs) was identified and all RBCs in that field were counted. This was repeated five times to calculate an average number of RBCs per field. One hundred fields, or the maximum number of viewable fields on the slide, were scanned for intra-erythrocytic parasites morphologically consistent with *Plasmodium*. Parasitaemia of positive smears was calculated following a previously described formula [20].

### DNA extraction, PCR, and Sanger sequencing

DNA was extracted from Whatman FTA Classic Cards following modified manufacturer’s instructions. Briefly, a 4-mm sample disc was punched from each FTA card and placed in a PCR amplification tube. FTA purification reagent (200 µl) was added to each tube and incubated for 5 min at room temperature. The FTA purification agent was removed by pipette, and this process repeated an additional three times. 200 µl of TE buffer (10 mM Tris-HCl, 0.1 mM EDTA, pH 8.0) was then added to each PCR tube and incubated for 5 min at room temperature. TE buffer was removed by pipette and this process was repeated once more. The sample disc was allowed to dry completely at room temperature before the disc was used as template in PCR amplification. This method resulted in consistently higher, better quality DNA yields than extraction methods that eluted DNA, such as *prep*GEM (ZyGEM NZ Ltd, Hamilton, New Zealand) or QIAamp DNA Investigator (QIAGEN, Hilden, Germany) kits.

A semi-nested PCR targeting the cytochrome b (cytb) gene of *Plasmodium* was conducted using primers CytB3384F (5′-GTAATGCCTAGACGTATTCCTG-3′) and CytB4595R (5′-GTTTGCTTGGGAGCTGTAATC-3′) in the external reaction, and CytB3706F (5′-GTTTGCTTGGGAGCTGTAATC-3′) and CytB4595R in the internal reaction. This procedure generated amplicons of 1254 bp (external) and 932 bp (internal) predicted size [20]. Both external and internal reactions were performed in 25 µl volumes using the FailSafe system (EpiCenter Biotechnologies, Madison, WI, USA), with reactions containing 1× FailSafe PCR PreMix with Buffer E, and 1 unit of FailSafe Enzyme Mix, 2.5 pmol of each primer. For external reactions, sample discs were used as template. For internal reactions, 1 µl of purified external PCR product were used as template. Reactions were cycled using previously described profiles [20]. A *Plasmodium gallinaceum*-positive sample that underwent the same extraction method was used as a positive control; exogenous DNA added post-DNA extraction was used as internal negative control (Qiagen, Hilden, Germany). All samples were run in duplicate at separate intervals to ensure diagnostic accuracy.

For samples identified as positive for *Plasmodium* by PCR but negative by microscopy and/or RDT, amplicons were Sanger sequenced in both directions using internal primers CytB3706F and CytB4595R on ABI 3730xl DNA Analyzers (Applied Biosystems, Carlsbad, CA) at the University of Wisconsin Biotechnology Center DNA Sequence Facility. Sequences were hand-edited using Sequencher v4.9 (Gene Codes Corporation, Ann Arbor, MI). To identify parasite species, newly generated DNA sequences were compared to reference sequences in Genbank using BLASTn [21].

### Analysis

The sensitivity and specificity of RDTs and microscopy were compared to PCR, considered the primary reference standard because it is established to outperform microscopy in sensitivity and specificity [22–24]. Parallel testing was also conducted, which combined the results of RDTs and microscopy. This determined if the combination of these two methods, which may be feasible to perform in larger healthcare facilities, can match the diagnostic accuracy of PCR, which is not realistic in most developing country healthcare settings.

## Results

A total of 78 patients were enrolled in this study (38 women and 40 men). All patients were tested by RDT. Blood samples for PCR were collected from 71 of these patients, and blood samples for microscopy were collected from 67 patients. Parasitaemia ranged from 0.003 to 3.399 % of total RBCs in *Plasmodium*-positive samples (as determined by microscopy). Average parasitaemia was 0.45 % (±1.00 SD) in females and 0.31 % (±0.43 SD) in males, and was 1.26 % (±1.43 SD) in children aged five or younger and 0.16 % (±0.31 SD) in patients older than five. Females presenting with fever were more frequently positive for malaria than males (41.2 % prevalence in females, 35.1 % in males by PCR; Table 1). In contrast, patients aged five or less were diagnosed with malaria at roughly equal frequency to those older than five (40.0 % prevalence in patients ≤5, 39.3 % prevalence in patients >5 by PCR; Table 1). Overall, the prevalence of malaria-positive patients was 38.0 % by the gold standard method, PCR (Table 1).

**Table 1:** Data summary

RDT				Microscopy				PCR			
M [40]	F [38]	≤5 [18]	ALL [78]	M [33]	F [34]	≤5 [15]	ALL [67]	M [37]	F [34]	≤5 [15]	ALL [71]
30.0	52.6	44.4	41.0	33.3	50.0	40.0	41.8	35.1	41.2	40.0	38.0

Data reflects the percentage of positive samples discovered by each diagnostic test in two patient demographics: sex (*M* male, *F* female) and age (≤5 = patients 5 years of age or less). Square brackets indicate sample sizes

In order to assess the efficacy of RDTs, the most promising diagnostic method in resource-limited regions, the sensitivity and specificity of RDTs and microscopy were compared to PCR. PCR identified 27 patients (out of 71) as positive for *Plasmodium*. Microscopic examination of peripheral blood smears identified 28 patients (out of 67) as positive for *P. falciparum*, and RDTs identified 32 patients as positive (out of 78; Table 1). Compared to PCR, RDTs were 92.6 % sensitive and 95.5 % specific, while microscopy was 92.0 % sensitive and 94.4 % specific (Table 2). Combining the results of RDTs and microscopy yielded 96 % sensitivity and 100 % specificity.

**Table 2:** Performance of three methods for malaria diagnosis

Diagnostic comparison	True+	True−	False+	False−	Sensitivity (95 % CI)	Specificity (95 % CI)
RDT vs. PCR	25	42	2	2	92.6 (75.6–98.9)	95.5 (84.5–99.3)
Microscopy vs. PCR	23	34	2	2	92.0 (73.9–98.8)	94.4 (81.3–99.2)
mDRT + microscopy vs. PCR	24	34	0	1	96.0 (79.6–99.3)	100 (89.6–100)

True readings indicate agreement between diagnostic methods. False readings indicate discrepancies between diagnostic methods. Test performance of microscopy and RDT were assessed relative to PCR as sensitivity (true positive rate) and specificity (true negative rate)

RDTs and microscopy both missed one patient sample that was positive by PCR. Interestingly, DNA sequencing revealed that this sample was 100 % identical to two published *Plasmodium malariae* cytochrome b sequences (Genbank Accession Numbers AB489194 (unpublished) and AB354570 [25]; BLASTn). Another sample was positive by PCR and microscopy (parasitaemia = 0.013 %) but negative by RDT. Sequencing identified this sample as 99 % identical to sequences from a published set of *P. falciparum* cytochrome b sequences from India [26]. Similarly, an additional sample positive by PCR and RDT but negative by microscopy was also determined to be *P. falciparum*, sharing 100 % sequence identity with sequences from the aforementioned Indian population set [26]. Lastly, two patients were negative by PCR but positive by RDT and microscopy.

## Discussion

This study evaluated the rate of malaria misdiagnosis occurring in rural Uganda, and assessed the accuracy of RDTs, the most promising diagnostic method for limiting over-diagnosis in resource-limited settings. All individuals in this study were symptomatically diagnosed with malaria by health practitioners, based on the presence of fever. However, results of the gold standard diagnostic test suggest that only 38.0 % of patients were positive for *Plasmodium*. Thus, the majority of patients (62.0 %) were misdiagnosed and would have been treated with anti-malarials if not for diagnostic intervention. These results corroborate findings of malaria over-diagnosis elsewhere. For example, a cross-sectional study evaluating Uganda’s policy of treating febrile illness with anti-malarials reported rates of over-diagnosis ranging from 45.3 to 80.9 % [27]. Indeed, malaria over-diagnosis is a well-known and widespread issue occurring throughout malaria endemic regions, with overestimates typically being greater than 30 % [1].

Results indicate that RDTs performed as well as the traditional gold standard, microscopy. Both were over 90 % sensitive and specific, although RDTs were marginally better at detecting infection when present and returning negative results when the parasite was absent. That RDTs are as effective or better than microscopy is consistent with the findings of others [28]. However, it should be noted that microscopy was performed under ideal conditions and by expert microscopists, which was necessary to establish accurate estimates of malaria misdiagnosis. A study that compared the diagnostic efficacy of RDTs to microscopy conducted in field conditions reported that RDTs had significantly higher sensitivity but lower specificity than microscopy [29].

Combining RDT and microscopic diagnosis resulted in perfect specificity, which indicates that malaria-negative patients are unlikely to be misdiagnosed as positive by combining these methods. However, one malaria-positive patient was still missed, even when RDTs and microscopy were combined. This patient was only detected by PCR, and sequencing revealed infection with *P. malariae*. In this study, RDTs specific for *P. falciparum* were selected based on this species’ ubiquity in the study area, and their recommended usage for malaria diagnosis in Uganda [30]. However, results from this study raise an important concern moving forward with malaria eradication efforts. Specifically, non-falciparum malaria patients face an elevated risk of misdiagnosis, even when recommended RDT diagnostic testing is applied. While microscopy is considered a better diagnostic tool for detecting non-falciparum malaria [28], this study’s results indicate that even a combinatorial approach does not result in infallible accuracy, especially when dealing with non-falciparum infection. Furthermore, the Uganda Malaria Strategic Reduction Plan (2014–2020) estimate the prevalence of non-falciparum species (2 % for *P. malariae* and *Plasmodium vivax*, <1 % for *Plasmodium ovale*) based on surveys published in the 1960s [31]. Needless to say, up-to-date surveys of non-falciparum malaria are necessary to estimate how frequently misdiagnosis of non-falciparum malaria might arise.

A surprising result in this study was that PCR failed to detect two samples that were confirmed positive by RDT and microscopy. Other studies have suggested that PCR false positives can be the result of recently cured malaria [24]. However, given that parasites were detected by microscopy in addition to RDT makes it possible that instead, PCR failed to detect parasites from these samples. Given that the percentage parasitaemia identified by microscopy were all within reasonable PCR detection limits, these two false-negatives may be a consequence of very limited blood quantities for DNA extraction, which highlights the necessity to fully saturate Whatman cards for accurate diagnosis. Since this issue is most likely to occur in young children where blood quantities from finger pricks is limited, RDTs (which require a single drop of blood) may in fact offer a performance advantage over PCR in this demographic.

Despite limitations in detecting non-falciparum malaria, the new generation of RDTs satisfy all criteria required for implementation in rural settings, being easy to use and durable over the long-term in tropical conditions [32]. Furthermore, a recent analysis estimated that RDTs are more cost-effective than microscopy per case correctly diagnosed and treated (at US$5.00, as compared to microscopy at US$9.61) [33]. Therefore, RDTs overall offer a promising approach to alleviate the costs of presumptive diagnosis in Africa’s high and medium–high transmission regions.

## Conclusions

Despite global efforts to eradicate malaria, the burden of this disease is still high. In rural Uganda, 38.0 % of patients visiting peripheral health clinics with fever were positive for malaria. However, this means that 62.0 % of patients symptomatically diagnosed with malaria were negative for the parasite, suggesting that over-diagnosis of this disease remains a critical problem. Combining both microscopy and RDT testing yielded high sensitivity and specificity. However, only PCR detected non-falciparum parasites, and is the only method that offers species-level diagnoses. Nevertheless, the cost and expertise required for this method render it impractical in nearly all developing healthcare settings. On the other hand, the ease and rapidity of RDTs confirm this method is perhaps the best approach for reducing malaria over-diagnosis, even in resource-limited settings.

## Abbreviations

PCR: polymerase chain reaction
RDT: rapid diagnostic test, refers specifically to test for *Plasmodium falciparum* malaria
